# Wavelength-Tunable, Ultra-Broadband, Biconical, Long-Period Fiber Grating Mode Converter Based on the Dual-Resonance Effect

**DOI:** 10.3390/s21175970

**Published:** 2021-09-06

**Authors:** Yu Zheng, Huiyi Guo, Mao Feng, Zhi Wang, Yange Liu

**Affiliations:** Tianjin Key Laboratory of Micro-Scale Optical Information Science and Technology, Institute of Modern Optics, Nankai University, Tianjin 300350, China; 2120190245@mail.nankai.edu.cn (Y.Z.); guohuiyi@mail.nankai.edu.cn (H.G.); nkufengmao@mail.nankai.edu.cn (M.F.); zhiwang@nankai.edu.cn (Z.W.)

**Keywords:** fiber grating, dual-resonance effect, mode converter, ultra-broadband

## Abstract

We demonstrated a wavelength-tunable, ultra-wideband, biconical, long-period fiber grating (BLPFG) mode converter in a two-mode fiber based on fusion taper technology and CO_2_ laser writing technology. Theoretical and experimental results show that after changing the diameter of the two-mode fiber by fusing and tapering, the dispersion turning point of the fiber is adjusted and wavelength-tunable broadband mode conversion is achieved efficiently. Theoretical simulation shows that the mode conversion bandwidth can cover the O + E + S + C band. In the experiment, we fabricated adiabatic tapers with cladding diameters of 113 μm and 121 μm and wrote gratings on these tapers to achieve dual-resonance coupling, thus realizing mode conversion from LP_01_ to LP_11_, with a 15 dB bandwidth of 148.8 nm from 1229.0 nm to 1377.8 nm and of 168.5 nm from 1319.7 nm to 1488.2 nm, respectively. As far as we know, this is the first time that fusion taper technology has been used to adjust the window of the dual-resonant coupling of an optical fiber. This work broadens the scope of application of the dual-resonance effect and proposes a general method for widening the bandwidth of a fiber grating with tunable wavelength.

## 1. Introduction

As one of the important components of guided-wave optical interconnection, mode converters come in various forms, such as spatial light modulators [1], binary phase plates [2,3], mode-selective couplers [4,5,6,7], fiber Bragg gratings [8,9,10,11], long-period fiber gratings [12,13,14,15], photonic lanterns [16,17,18,19,20], and multi-planar optical converters [21]. According to their physical structure, they can be divided into three categories: free-space-based converters, optical fiber-based converters, and waveguide-based mode converters. Free-space-based mode converters suffer from a high insertion loss and large size. Waveguide-based mode converters are complicated to manufacture. As a comparison, optical fiber-based mode converters are attractive due to their compact design, low insertion loss, and compatibility with optical fiber communication networks. Fiber-based mode converters, such as long-period fiber gratings (LPFGs), can be manufactured by mechanical micro-bending [12], carbon dioxide laser writing [13], and acoustic induction [15,22].

For decades, fiber gratings have received widespread attention due to their ease of preparation and high efficiency. So far, linearly polarized (LP) mode conversion from LP_01_ to LP_11_, LP_21_, LP_31_, and LP_02_ using a fiber grating has been realized [23,24,25]. However, the fiber grating suffered from its narrow bandwidth due to the inherent influence of the working mechanism. A lot of work has been devoted to expanding the working bandwidth of fiber gratings [5,10,11,17,26]. Rottwitt used chirped gratings to increase the 20 dB mode conversion bandwidth by 4.8 times (8.6 nm) [27]. Zhao et al. cascaded three linear, length-apodized, phase-shifted, long-period gratings to achieve a 10 dB bandwidth of 182.0 nm [28]. Guo et al. used the double-resonance effect to obtain a 15 dB bandwidth of 118.2 nm [29]. The performance comparison of these mode converters is shown in Table 1. Compared with other methods, the dual-resonance effect does not need to design a complicated grating structure, nor does it require a special processing technology. It is the simplest method with the best performance. However, the dual-resonance effect can only broaden the bandwidth of the grating at a specific wavelength range determined by the fiber parameters, which limits the application of the dual-resonance effect.

In this paper, we demonstrated the production of a wavelength-tunable, ultra-wideband, biconical, long-period fiber grating (BLPFG) mode converter in a two-mode fiber based on fusion taper technology and CO_2_ laser writing technology. Theoretical and experimental results show that there is dual-resonance coupling at the dispersion turning point of the two-mode fiber, which effectively expands the working bandwidth of the grating. By changing the diameter of the fiber by fusing and tapering, the dispersion turning point of the fiber is adjusted and broadband mode conversion is achieved efficiently. Theoretical simulation shows that the mode conversion bandwidth can cover the O + E + S + C (O: 1270~1370 nm, E: 1370~1460 nm, S: 1460~1530 nm, C: 1530~1565 nm) band. In the experiment, we made adiabatic tapers with cladding diameters of 113 μm and 121 μm and wrote gratings on the taper to achieve dual-resonance coupling. The fabricated BLPFGs realized the mode conversion from LP_01_ to LP_11_ with a 15 dB of 148.8 nm (1229.0~1377.8 nm) and 168.5 nm (1319.7~1488.2 nm), respectively. The size of the bandwidth can be adjusted by changing the grating period so as to realize the large-bandwidth mode conversion with adjustable wavelength. This work broadens the scope of application of the dual-resonance effect and proposes a general method for widening the bandwidth of a fiber grating with tunable wavelength.

## 2. Theory and Simulation Results

**Figure 1:**
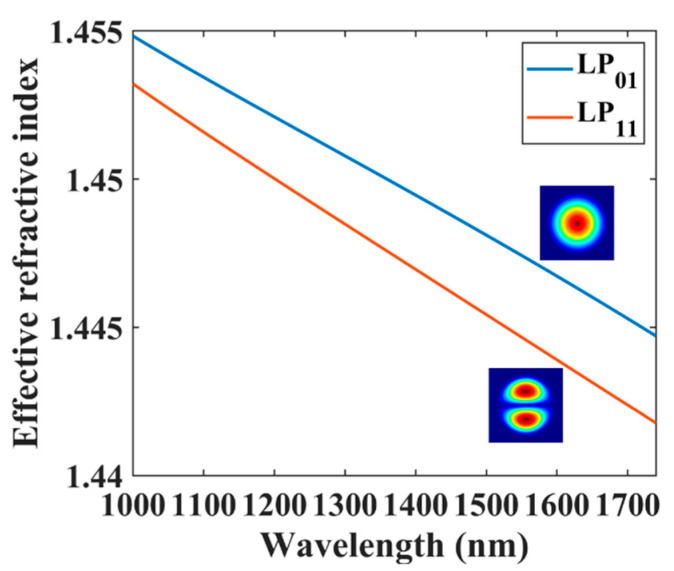
Dispersion curve of the TMF.

The fiber used in this work was a step-index two-mode fiber (TMF) (SM2000, Thorlabs) with a core diameter of 11 μm and a cladding diameter of 125 μm. The numerical aperture (NA) of the fiber is 0.12. We used the finite element method (FEM) to simulate the fiber mode dispersion curves, and the results are shown in Figure 1. The results show that the fiber supports LP_01_ and LP_11_ modes from 1000.0 nm to 1750.0 nm. The effective index difference of the two modes was above 10^−4^ in the simulation wavelength range. Thus, the fiber was used as a robust TMF in this work.

Coupled mode theory [30] demonstrates that the grating period of an LPFG is inversely proportional to the effective index difference between the fundamental mode and the target mode:

(1) $$\Lambda =\frac{\lambda }{n_{eff,01}-n_{eff,11}\;\;}$$

where Λ is the period of the LPFG, *λ* is the resonant wavelength of the LPFG, and $n_{eff,01}$ and $n_{eff,11}$ are the effective refractive index of LP_01_ and LP_11_ modes, respectively.

The coupling coefficient of LP_01_ and LP_11_ modes can be expressed as a function of the refractive index modulation $\Delta \varepsilon_{q}$,

(2) $$\kappa =\frac{\omega }{4}\iint_{-\infty }^{\infty }{\vec{E}}_{01}^{*}\Delta \varepsilon_{q}{\vec{E}}_{11}ds$$

where ω is the circular frequency of the light wave and ${\vec{E}}_{01}$ and ${\vec{E}}_{11}$ are the normalized mode fields of LP_01_ and LP_11_, respectively_._ Then the transmission spectrum of the LP_01_ mode of an LPFG is [29]

(3) $$P_{01}\left(z\right)=1-\frac{\pi^{2}}{\pi^{2}+4L_{c}^{2}\delta^{2}}\sin^{2}\left(L_{c}\sqrt{\frac{\pi^{2}}{4L_{c}^{2}}+\delta^{2}}\right)$$

where the coupling length $L_{c}=\pi /\left(2\left|\kappa \right|\right)$ and the parameter of phase mismatch $\delta =\frac{1}{2}\left[\beta_{01}-\left(\beta_{11}+q\frac{2\pi }{\Lambda }\right)\right],q=1,2,3,\ldots$ In general, we set q=1.

**Figure 2:**
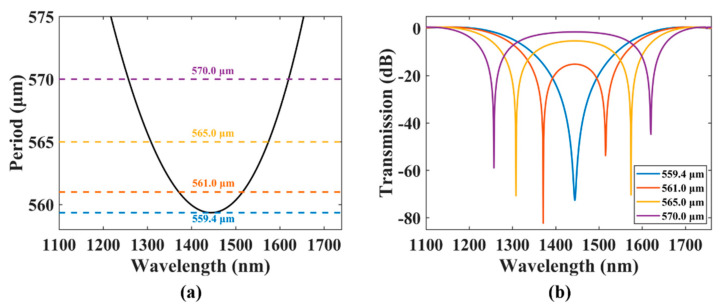
(**a**) The LPFG period versus the wavelength variation curve. (**b**) The LPFG transmission spectra with different periods.

We calculated the period of the TMF-LPFG versus different wavelengths using Equation (1), and the results present a non-monotonic variation trend of parabola, as illustrated in Figure 2a. The LPFG period first fell and then rose as the wavelength increased, and reached the minimum of 559.4 μm at a wavelength of 1444.0 nm, which is called the dispersion turning point. That is, one grating period value corresponds to two resonant wavelengths when the grating period is larger than the value at the dispersion turning point. While the grating period is near the dispersion turning point, the two resonant wavelengths get close and merge with each other, and then the bandwidth that satisfies the mode conversion condition doubles [29,31].

Figure 2b shows the simulated spectra of the LPFGs with different periods marked in Figure 2a according to Equation (3). The spectrum lines and their corresponding period markers share the same color. When the period was 570.0 μm as the purple lines show, the two resonance wavelengths were 1257.0 nm and 1620.0 nm and the resonance dips appeared at the two wavelengths in the spectrum, and their 15 dB bandwidths were 29.0 nm and 26.0 nm, respectively. When the period was reduced, as the orange and yellow lines indicate, the two resonance dips gradually moved closer and touched each other, and the 15 dB bandwidth reached the maximum value of 204.0 nm when the period was 561.0 μm. When the period was further reduced to 559.4 μm (blue line), the two dips merged into a broadband single dip with a 15 dB bandwidth of 144.0 nm. Therefore, to achieve the largest-possible bandwidth, a period slightly larger than the corresponding dispersion turning point should be selected. The detailed situation mentioned above is shown in Table 2.

Now we can only achieve broadband mode conversion around 1444.0 nm. The reason is that the dispersion turning point of the optical fiber depends on the inherent structure of the optical fiber. For any kind of few-mode fiber, the dispersion turning point corresponding to the desired mode conversion is fixed, that is, we cannot take advantage of the dual-resonance effect at a wavelength away from the dispersion turning point.

The key is to change the optical structure of the fiber. Equation (1) shows that the grating period Λ is a function of the mode effective index $n_{eff,01}$ and $n_{eff,11}$. If we change the geometric parameters of the fiber, the value of $n_{eff,01}-n_{eff,11}$ also changes. Thus, the curve of the grating period Λ in the wavelength domain deforms and the turning point changes its position. In other words, the dispersion turning point moves in the wavelength domain by changing the structure of the optical fiber so that the dual-resonance effect can be used in other bands. As the simplest method, fusing and tapering technology is chosen to change the diameter of the core and cladding. When the fiber is stretched to a certain ratio, the characteristics of gratings with different periods can be simulated by the same method.

**Figure 3:**
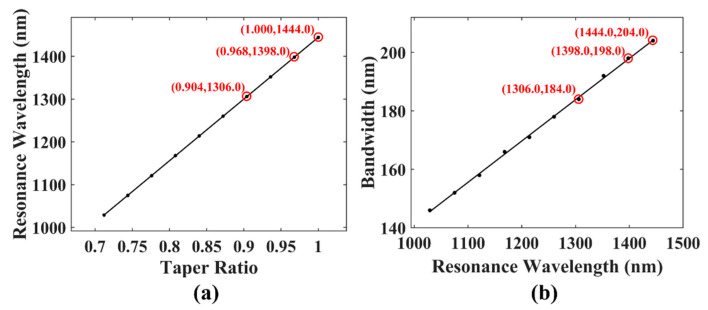
(**a**) The change in the dispersion turning point with the taper ratio. (**b**) The change in the 15 dB bandwidth with the dispersion turning point.

Here we focused on the influence of different stretching ratios on the dispersion turning points and grating bandwidths. As shown in Figure 3a, as the diameter of the fiber became smaller, the dispersion turning point moved from the initial value of 1444.0 nm to the shortwave direction. The change in the dispersion turning point along the fiber diameter was almost linear, and the R-square (determination coefficient) of the linear fit was 1.0000. The movement of the dispersion turning point also caused a change in the bandwidth, as shown in Figure 3b. The bandwidth of the grating decreased when the dispersion turning point blue-shifted. Similarly, the change was linear, and the determination coefficient was 0.9992. The simulation results show that the position of the dispersion turning point can be adjusted by tapering the fiber. For the fiber used in this work, its dispersion turning point moved from the initial value of 1444.0 nm to the shortwave direction and its mode conversion bandwidth could cover the O + E + S + C band by adjusting the diameter of the fiber.

**Figure 4:**
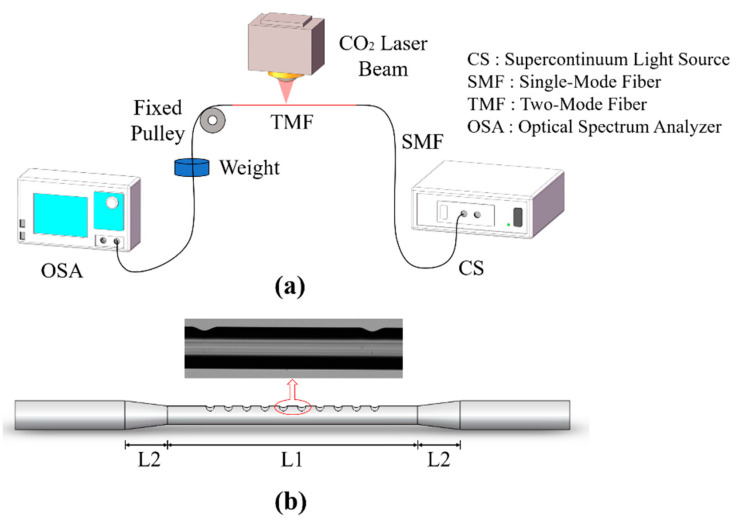
(**a**) Schematic diagram of the BLPFG preparation. (**b**) Schematic diagram of the BLPFG and micrograph of one period in the taper waist.

The marked points in Figure 3 corresponding to the cladding diameter of 125 μm, 121 μm, and 113 μm were then taken into the experiment to achieve dual-resonance broadband mode conversion surrounding 1444.0 nm, 1398.0 nm, and 1306.0 nm, respectively. The fiber grating fabrication process included two steps. First, the optical fiber was stretched adiabatically to the desired size. The equipment used for tapering was a Vytran GPX 3850 Glass Processor Workstation. The flame scan width was set to 20 mm, and the stretched length was 2.00 mm and 0.66 mm for the final diameter of 113 μm and 121 μm, respectively. The obtained fiber taper was composed of a waist with a length of L1 and a taper area with a length of L2, as shown in Figure 4b. For the fabricated taper in this work, L1 was 20 mm, which is long enough for the fiber grating fabrication. L2 was approximately equal to the stretching length of the fiber, which is 2.00 mm and 0.66 mm, respectively. Real-time power monitoring during processing shows that the fiber taper is flat enough to ensure a low transmission loss below 0.1 dB.

After obtaining the adiabatic taper, we used a CO_2_ laser (Han’s CO_2_-H30) to process the long-period grating at the waist of the taper. The processing device is shown in Figure 4a. Both ends of the adiabatic taper were fused to a single-mode fiber (SMF). A supercontinuum light source (SC-5-FC 480–2200 nm) and a spectrum analyzer (YOKOGAWA AQ6370D) were connected to the ends of the SMF to monitor the transmittance of the fundamental mode in the BLPFG. A 15 g counterweight was attached to the optical fiber to ensure constant tension during processing. The taper waist was single-side-exposed by the CO_2_ laser pulse with a power of 2.1 W. When the optical fiber was irradiated, the glass melted and deformed slightly under the action of tension, forming a refractive index modulation. Usually, the fiber needs to be exposed repeatedly several times to achieve the best performance. Figure 4b shows a schematic diagram of the obtained BLPFG and a photomicrograph of one period in the taper waist. The CO_2_ laser side irradiation formed a trench with a depth of 10.0 nm and a width of 50.0 nm.

## 3. Results and Discussion

**Figure 5:**
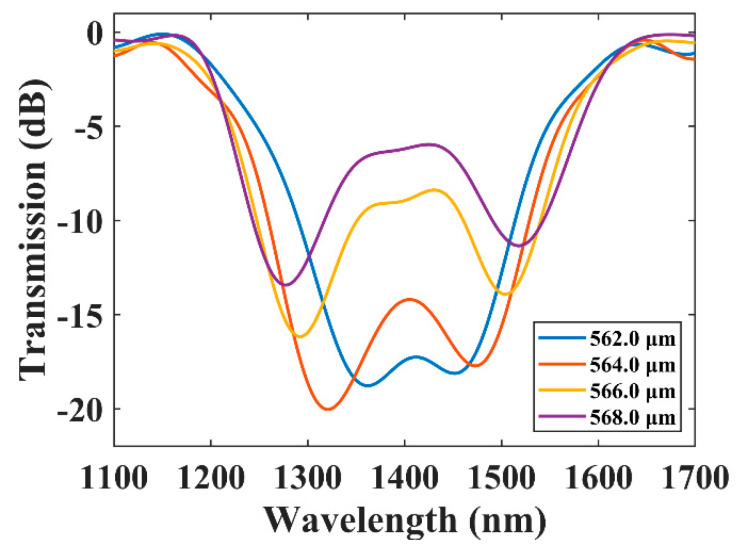
The transmission spectra of BLPFGs. The cladding diameter is 121 μm, and the periods are 568.0 μm, 566.0 μm, 564.0 μm, and 562.0 μm.

We first confirmed that the dual-resonance effect is still effective on the tapered fiber. Figure 5 shows the transmission spectra of the BLPFGs with a cladding diameter of 121 μm and periods of 568.0 μm, 566.0 μm, 564.0 μm, and 562.0 μm. Each spectrum was filtered by FFT to remove the slight interference of LP_01_ and LP_11_ caused by the fusion of SMF and TMF. There were two resonance dips when the period was 568.0 μm. As the grating period decreases, the two resonant dips approach each other gradually, and the bridge between the two dips decreases. When the period was reduced to 562.0 μm, the two dips combined into a broadband dip and the 15 dB bandwidth reached the maximum of 168.5 nm from 1319.7 nm to 1488.2 nm. Thus, as the period decreases, the 15 dB bandwidth increases. The experimental spectra show the same change trend as the simulation results in Figure 2b.

**Figure 6:**
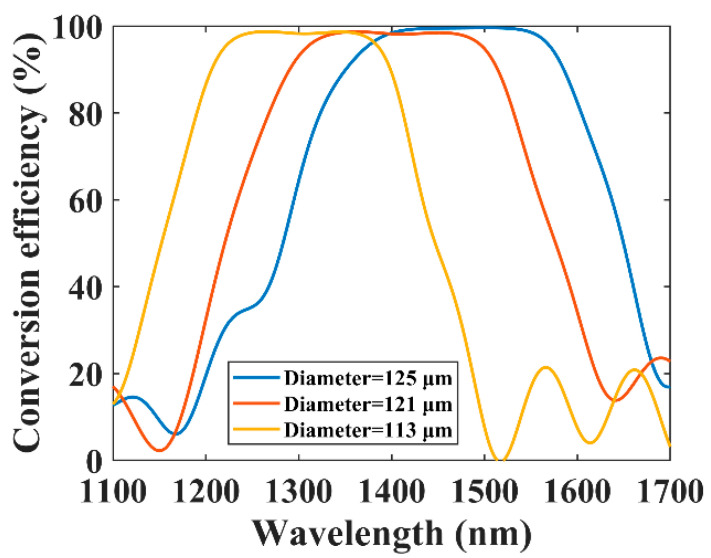
The conversion efficiency of three BLPFGs with a diameter of 113 μm, 121 μm, and 125 μm, respectively.

The origin dispersion turning point of the used fiber was located at 1474.0 nm. After tapering to 121 μm and 113 μm, we moved the dispersion turning point to a shorter wavelength and realized broadband mode conversion. Figure 6 shows the conversion efficiency of the BLPFGs with a diameter of 113 μm, 121 μm, and 125 μm. When the cladding diameter of the fiber was 125 μm, the 15 dB (corresponding to a conversion efficiency of 96.8%) bandwidth was 180.0 nm from 1383.7 nm to 1563.7 nm and the 20 dB (corresponding to a conversion efficiency of 99.0%) bandwidth was 130.2 nm from 1412.5 nm to 1542.7 nm. The center wavelength of the band was 1474.0 nm. When the fiber was stretched to 121 μm, the 15 dB bandwidth was 168.5 nm from 1319.7 nm to 1488.2 nm. The center wavelength reached 1404.0 nm and a blue shift occurred. The situation was similar when the fiber diameter was further reduced to 113 μm. The 15 dB bandwidth was 148.8 nm from 1229.0 nm to 1377.8 nm, and the center wavelength moved to 1303.4 nm. The experimental results show a blue shift of the dispersion turning point and a decrease in the 15 dB bandwidth when the taper ratio decreases. The experimental results are in good agreement with the simulation results. It should be noted that compared with the theoretical results, the dispersion turning point of the BLPFG slightly changed and the 15 dB bandwidth reduced in the experiment. This may come from the measurement error of the fiber size. The fabricated BLPFGs have a wide bandwidth and can overlap a part of each other, so the LP_01_-to-LP_11_ mode conversion from 1000.0 nm to 1550.0 nm is realized. The comparison between our experimental results and the other ones used to expand the mode conversion bandwidth is shown in Table 3.

**Figure 7:**
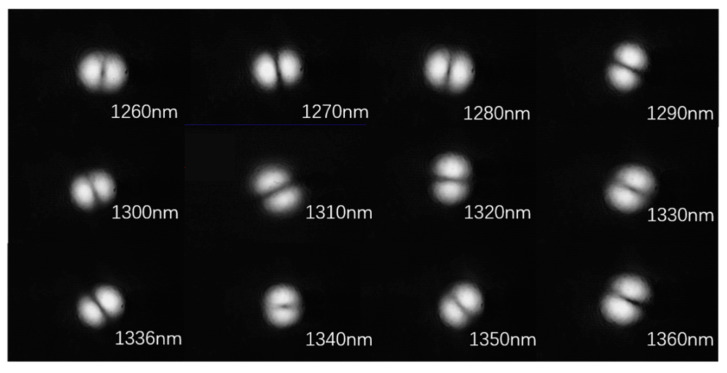
The output modes of the BLPFG in its working window.

We observed the output mode field of the fabricated BLPFG with a cladding diameter of 113 μm and a period of 528.0 μm. The obtained mode field by a CCD camera is shown in Figure 7. The clear two-lobed LP_11_ mode field indicates that the mode converter has a high conversion efficiency.

## 4. Conclusions

In summary, we proposed an ultra-broadband BLPFG mode converter based on the dual-resonance coupling effect. By changing the fiber cladding diameter, we moved the dispersion conversion point from 1474.0 nm to 1404.0 nm and 1303.4 nm and achieved working bandwidths of 168.5 nm and 148.8 nm, respectively. Since the diameter of the fiber taper can be made according to the parameters we need, the dispersion turning point can be adjusted to the desired position. Besides, the mode conversion bandwidth can be adjusted by changing the grating period. In this way, dual modulation of the dispersion turning point and the mode conversion bandwidth can be achieved. The proposed BLPFG broadens the scope of application of the dual-resonance effect and proposes a general method for widening the bandwidth of a fiber grating with tunable wavelength.

## Figures and Tables

**Table 1:** The performance difference between the three mode converters.

Methods	Bandwidth Adjustability	Working Band Adjustability	Shortcoming
Chirped gratings	Yes	No	The introduction of chirp in an LPFG will cause a reduction in the coupling efficiency.
Three linear-length apodization profiles to cascade	Yes	Yes	The conversion rate is not high enough.
The double-resonance effect	Yes	No	Mode conversion can only be achieved in the fixed band.

**Table 2:** Characteristic parameters of simulated spectra corresponding to different periods of the LPFG.

Period (μm)	15 dB Bandwidth (nm)		Bandwidth Range (nm)		Dip Position (nm)	
	Dip-L	Dip-R	Dip-L	Dip-R	Dip-L	Dip-R
570.0	29.0	26.0	1243.0–1272.0	1606.0–1632.0	1257.0	1620.0
565.0	41.0	37.0	1289.0–1330.0	1554.0–1591.0	1308.0	1574.0
561.0	204.0		1340.0–1544.0		1371.0	1515.0
559.4	144.0		1371.0–1515.0		1444.0	

**Table 3:** Data comparison of different methods to increase the mode conversion bandwidth in recent years.

Author, Year	Methods	Bandwidth	Band/Central Wavelength	Bandwidth Adjustability	Working Band Adjustability
Zhao et al., 2016	Tilted uniform LPFG [13]	20 dB, 17.8 nm	1507.0 nm	Yes	Yes
Rottwitt, 2016	Chirped gratings [27]	20 dB, 8.6 nm	800.0 nm	Yes	No
Wang et al., 2017	Length-apodized, long-period grating fabricated on a waveguide [32]	20 dB, 150 nm	-	Yes	No
Guo et al., 2018	Reduction in the number of grating periods [33]	15 dB, 76.0 nm	1567.2 nm	Yes	No
Zhao et al., 2019	Cascading three linear length-apodized gratings [28]	10 dB, 182.0 nm	C + L	Yes	Yes
Guo et al., 2019	The double-resonance effect [29]	15 dB, 118.2 nm	1000.0 nm	Yes	No
Zhang et al., 2019	Chiral LPFGs [34]	10 dB, 25.0 nm	-	-	-
This work	BLPFG	15 dB, 148.8 nm	1303.4 nm	Yes	Yes
		15 dB, 168.5 nm	1404.0 nm		
		15 dB, 180.0 nm; 20 dB, 130.2 nm	1474.0 nm		

## Data Availability

Not applicable.
